# Kikuchi-Fujimoto Disease: An Atypical Presentation of a Rare Disease

**DOI:** 10.7759/cureus.3999

**Published:** 2019-02-01

**Authors:** Amanda Moyer, Muhammad Z Hanafi, Teresa Scordino, Michael Bronze

**Affiliations:** 1 Pediatrics, University of Oklahoma Health Sciences Center, Oklahoma City, USA; 2 Gastroenterology, University of Oklahoma Health Science Center, Oklahoma City, USA; 3 Pathology, University of Oklahoma Health Sciences Center, Oklahoma City , USA; 4 Internal Medicine, University of Oklahoma Health Sciences Center, Oklahoma City, USA

**Keywords:** kikuchi fujimoto, lymphadenopathy, histiocytosis, lymphadenitis, epstein-barr

## Abstract

Kikuchi-Fujimoto disease (KFD), or necrotizing histiocytic lymphadenitis, is a rare cause of lymphadenopathy and fever. Although the clinical course is usually benign, KFD is often mistaken for malignancy or infection. Recognition of typical and atypical cases of KFD is necessary to avoid unnecessary interventions. Here we report an atypical presentation of KFD with diffuse lymphadenopathy and leukocytosis associated with high levels of circulating Epstein-Barr viral DNA.

## Introduction

Kikuchi-Fujimoto disease (KFD) is a benign lymphohistiocytic disorder characterized by lymphadenopathy (typically cervical), fatigue, and fever [1-2]. Additional common findings include hepatosplenomegaly, weight loss, elevated acute phase reactants, and leukopenia/lymphopenia [3].

The etiology of KFD is controversial. Some authors speculate that viral and autoimmune processes influence the development of KFD [4-5]. It generally resolves in one to four months, but has been associated with subsequent development of systemic lupus erythematous (SLE) [3, 5-6]. Excisional lymph node biopsy with hallmark findings confirms the diagnosis [7-8].

## Case presentation

A 20-year-old Caucasian female was admitted with one month of cervical lymphadenopathy and two months of fever, fatigue, night sweats, and 15-pound weight loss. A course of antibiotics two weeks prior did not improve her symptoms. A week prior to admission, she developed an intermittent diffuse urticarial rash.

Medical history was significant for poorly controlled type II diabetes and chronic pain. She was allergic to sulfa drugs and latex. Family history was unknown. She denied travel, drug and alcohol use, and sexual activity.

On presentation, the patient was febrile to 38.6^o^C, with a heart rate of 135 beats per minute, respiratory rate of 18 breaths per minute, and blood pressure of 115/85 mmHg. Examination was remarkable for diffuse, rubbery lymphadenopathy (0.5 cm × 1 cm–3 cm × 3 cm). A faint erythematous, reticular rash was present on her legs.

Laboratory data demonstrated marked leukocytosis with eosinophilia. Inflammatory markers, uric acid, and lactate dehydrogenase were elevated (Table 1).

**Table 1:** Laboratory data.

Variable	Reference range	Day 0	Day 3	Day 6	Day 23
					(Outpatient)
Hematology					
Hematocrit (%)	34.0-46.0	37.5	35.4	31.6	29.1
Hemoglobin (g/dL)	12.0-16.0	12.7	11.8	10.5	10
White blood cell count (K/mm^3^)	4.000-11.00	47.70	24.38	12.02	10.66
Differential (%)					
Neutrophils	39-78	23	39	38	
Band forms	0.0 - 12	16	2.0	9.0	-
Lymphocyte	15.0-46.0	34 (6% reactive)	22.3	24.0	38.1
Monocytes	2.0-14	4.0	1.0	1.0	4.6
Eosinophils	0.0-6.0	21	26	23	7.0
Basophils	0-2	1	2	2	-
Platelets (K/mm^3^)	140-440	126	114	183	388
Mean corpuscular volume (fL)	80.0-99.0	80.6	81.9	84	85.3
Red cell distribution width (%)	11.0-15.0	15.2	15.6	15.8	19.4
Westergren (mm/h)	0.0-20	93	-	110	-
Haptoglobin (mg/dL)	38.0-195	276	-	-	-
Smear description	Leukocytosis with neutrophilia, lymphocytosis with many reactive appearing or variant lymphocytes, plasma cells, and marked eosinophilia. Anemia with mild microsytosis. Thrombocytopenia, mild. No blasts. No overtly malignant cells or evidence of hemolytic process.				
Chemistry					
Sodium (mEq/L)	136-145	130	137	137	137
Potassium (mEq/L)	3.5-5.1	3.9	3.4	4.1	3.9
Chloride (mEq/L)	97-109	97	107	109	103
Carbon dioxide (mEq/L)	23-32	23	28	24	25
Blood urea nitrogen (mg/dL)	6-17	23	14	9	9
Creatinine (mg/dL)	0.7-1.1	1.25	0.8	0.73	0.9
Glucose (mg/dL)	66-111	104	154	143	148
Calcium (mg/dL)	8.7-10.1	9.1	8.3	8.6	9.3
Phosphorus (mg/dL)	2.5-4.5	1.7	2.7	4.3	-
Magnesium (mg/dL)	1.6-2.6	2.3	1.7	1.6	-
Uric acid (mg/dL)	2.6-6.0	9.8	5.8	2.9	-
Total bilirubin (mg/dL)	0.3-1.2	0.7	-	0.4	0.7
Aspartate aminotransferase (units/L)	8-41	22	-	20	21
Alanine aminotransferase (units/L)	12-48	23	-	19	22
Alkaline phosphatase (units/L)	55-145	85	-	89	83
Lactate dehydrogenase (units/L)	112-236	385	-	-	186
C-reactive protein (mg/L)	0.2-8.0	90	-	4.5	-
Total protein (g/dL)	6.1-7.7	9	8.4	0	9.9
Albumin (g/dL)	3.8-5.1	3.5	2.8	2.8	3.4
Ferritin (ng/dL)	10-322	-	254.2	-	-
Thyroid stimulating hormone (mIU/L)	0.350-4.940	2.491	-	-	-

Blood smear demonstrated reactive lymphocytes without circulating blasts. Flow cytometry did not show any abnormal lymphoid populations. Extensive infectious work up was negative, with the exception of Epstein-Barr virus (EBV) polymerase chain reaction (PCR) (Table 2). 

**Table 2:** Serology.

Test	Reference range	Day 0
Epstein-Barr virus PCR (IU/mL)	Not detected	33,600
Rapid plasma reagin	Nonreactive	Nonreactive
Cytomegalovirus DNA PCR	Not detected	Not detected
Human immunodeficiency virus 1&2 antibody	Nonreactive	Nonreactive
Herpes simplex virus 1&2 PCR	Not detected	Not detected
QuantiFERON®-TB Gold	Negative	Negative
Toxoplasma gondii DNA PCR	Not detected	Not detected
Hepatitis A IgM	Nonreactive	Nonreactive
Hepatitis B panel**	Nonreactive	Nonreactive
Hepatitis C antibody	Nonreactive	Nonreactive
**Hepatitis B panel includes surface antigen, surface antibody, core antibody, core IgM antibody		

**Figure 1:**
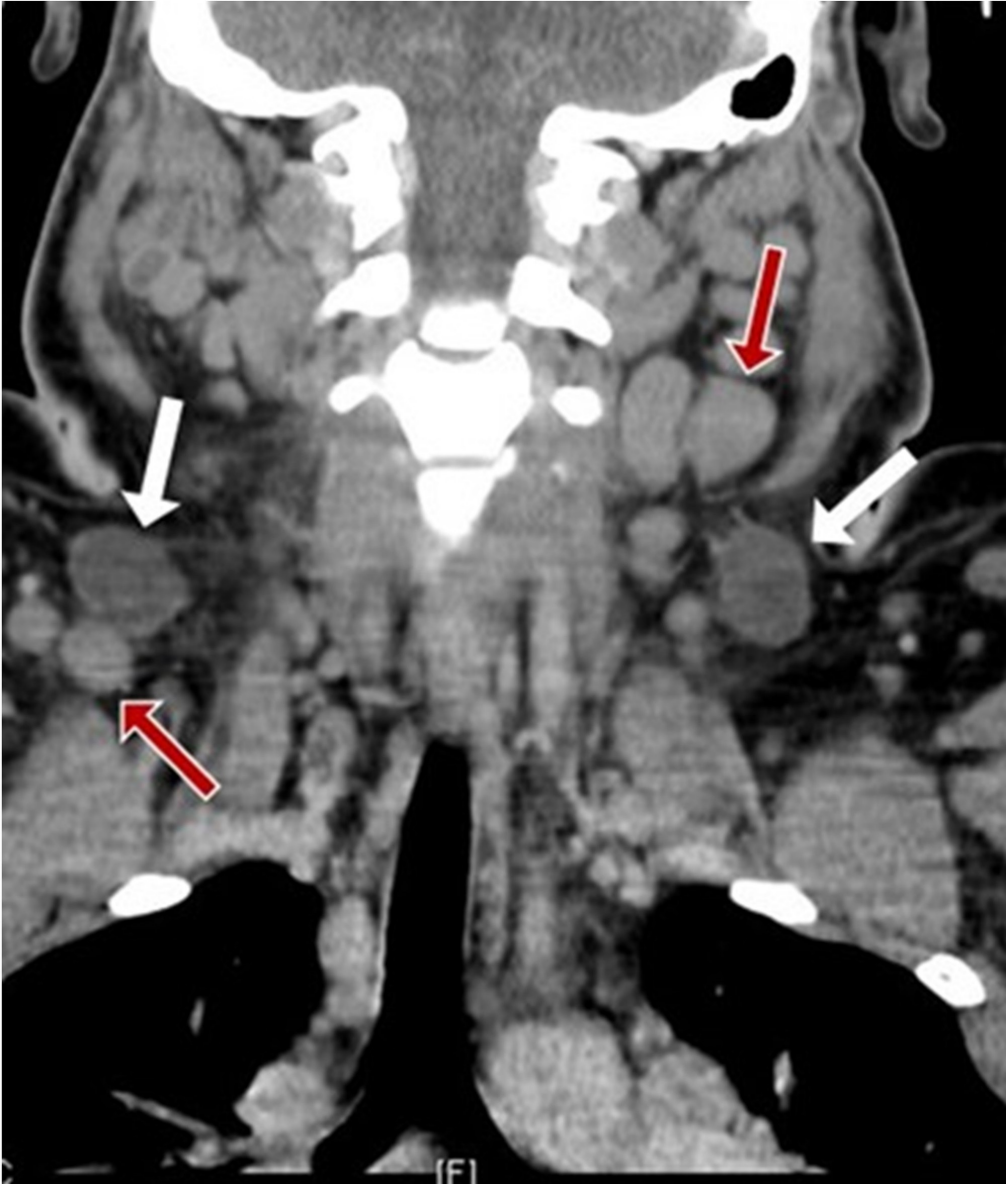
Computed tomography (CT) of the neck and chest. Marked cervical, supraclavicular, mediastinal, and upper abdominal lymphadenopathy and hepatosplenomegaly. Centrally necrotic cervical lymph nodes (white arrows) are compared to enlarged, but non-necrotic, lymph nodes (red arrows).

**Figure 2:**
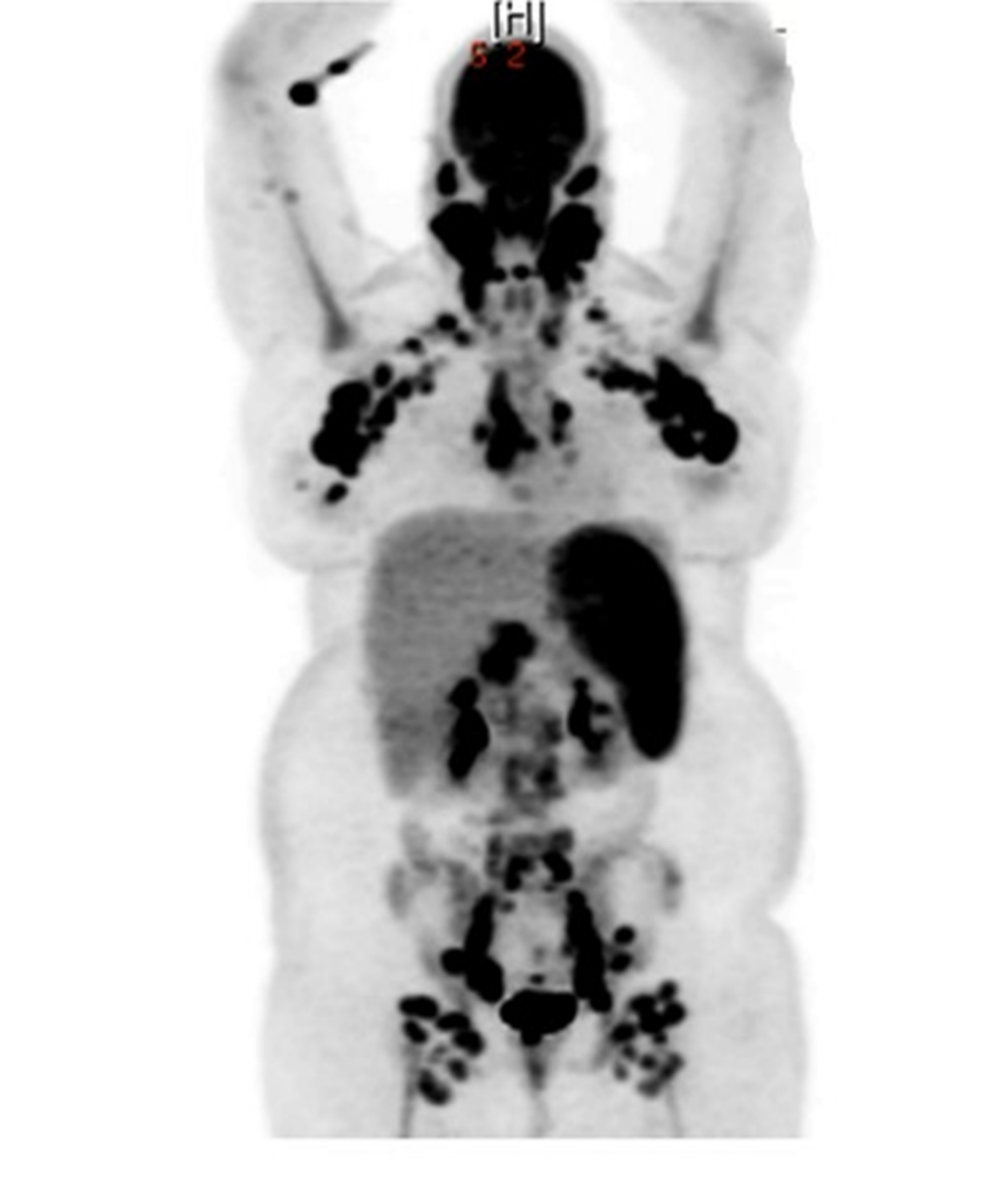
Positron emission tomography (PET) scan. Widespread hypermetabolic bulky lymphadenopathy. Standardized uptake values (SUVs) ranged from 9.9 to 15.0. Mild diffuse bone marrow uptake was present, but no hypermetabolic osseous lesions were identified. Spleen appeared to be involved with SUV up to 7.0.

A computed tomography (CT) scan (Figure 1) revealed marked lymphadenopathy and hepatosplenomegaly. Positron emission tomography (PET) imaging (Figure 2) showed widespread hypermetabolic bulky lymphadenopathy and diffuse bone, spleen, and marrow uptake without osseous lesions.

**Figure 3:**
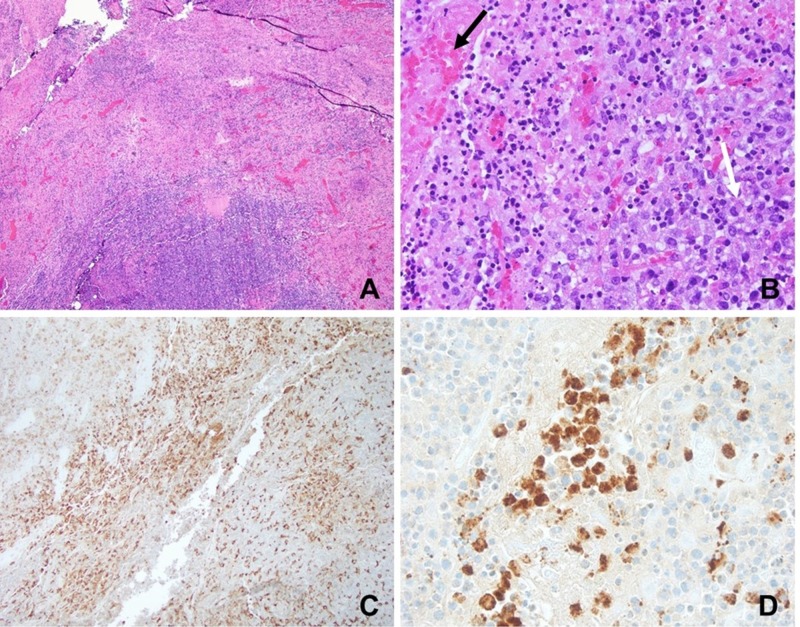
Excisional lymph node biopsy. (A) Extensive necrosis (pink), 40x. (B) Necrosis with karyorrhectic debris (black arrow) adjacent to sheets of histiocytes (white arrows). (C) CD68 immunohistochemical stain highlighting increased histiocytes in brown. (D) Myeloperoxidase-positive histiocytes (brown).

Excisional lymph node biopsy of a submental node revealed necrotizing lymphadenitis characterized by immunoblasts, myeloperoxidase-positive histiocytes with crescent shaped nuclei, and extensive necrosis with karyorrhectic debris (Figure 3).

There was no eosinophilic or neutrophilic infiltrate, or evidence of malignancy on biopsy or flow cytometry. Stains for acid-fast bacteria, fungi, herpes simplex virus, and cytomegalovirus were negative. Autoimmune workup was unrevealing (Table 3).

**Table 3:** Immunological studies.

Variable	Reference range	Presentation	Day 23 (Outpatient)
Rheumatoid factor screen (u/mL)	<10	<10	-
Cyclic citrullinated peptide Ab (CCP)	Negative	Negative	-
Anti-nuclear antibody (ANA)	Negative	Negative	Positive
		<1:40	1:120
Reichlin panel**	Negative	Negative	-
Anti-neutrophil cytoplasmic antibody	<1:20	1:40	<1:20
Complement C3	83.0-157	123	160
Complement C4	13-35	2.4	26
**Reichlin profile includes: ANA, dsDNA, anti-Sm, anti-nRNP, anti-Scl-70, anti-Ro/SSA, anti-La/SSB, anti-ribosomal-P, anti-Jo-1, anti-PM-Scl, and anti-Mi-2.			

The patient’s hospital course was uneventful. She received three doses of ceftriaxone for urinalysis findings consistent with urinary tract infection and was treated symptomatically with analgesics and anti-histamines. The patient’s fever, rash, and lab abnormalities resolved spontaneously by day 5 of hospitalization and her lymphadenopathy decreased dramatically by discharge.

## Discussion

Our case highlights a unique presentation of KFD in the setting of EBV infection. Diffuse lymphadenopathy, while rare, has been reported. To our knowledge, KFD has not been associated with the degree of leukocytosis or plasma cells on biopsy seen in our patient. These findings coupled with high titer EBV PCR and negative ANA suggest that EBV influenced the development of KFD in our patient.

Presentation

Although excisional lymph node biopsy is imperative for KFD diagnosis, clinical presentation and course support the diagnosis. KFD occurs most frequently in Asian populations, but has a worldwide distribution. KFD typically presents prior to the fourth decade, but there have been cases in pediatric and elderly populations. Female to male ratio ranges from 1:1 to 4:1, depending on the cohort [6, 8-11].

Kikuchi-Fujimoto disease presents acutely or sub-acutely. Common findings include cervical lymphadenopathy, fever, headache, weight loss, fatigue and nonspecific viral-like symptoms. Nonspecific skin manifestation (rashes, nodules, and erythema multiforme), hepatosplenomegaly, and diffuse lymphadenopathy may be present and likely indicate more severe disease [3, 6, 8, 10]. In a review of 91 cases consisting of predominately non-Asian females with KFD, Dumas found that 52% of patients exhibited generalized lymphadenopathy which is a significantly higher proportion compared to previous Asian predominant cohorts suggesting that KFD may present differently in non-Asian populations [2-3, 10].

Common laboratory findings include elevated inflammatory markers, leukopenia/lymphopenia, anemia, atypical lymphocytes, thrombocytopenia, and increased lactate dehydrogenase and alanine aminotransferase. Leukocytosis has been reported in 2%-5% of cases [6, 9-10, 12]. To our knowledge, the degree of leukocytosis, especially the eosinophilia demonstrated by our patient, has not been reported (Table 4). 

Diagnosis

**Table 4:** Characteristic findings in Kikuchi-Fujimoto disease based on lymph node biopsy.

Characteristic findings in Kikuchi-Fujimoto disease
Focal lesions situated in the lymph node cortex or paracortex
Severe coagulative necrosis with karyorrhexis
Absence of large numbers of large numbers of neutrophils and eosinophils (plasma cells are also typically scarce)
Histiocytes at the margin of necrotic areas (possibly foamy or crescent shaped)
Variable plasmacytoid monocytes, lymphocytes, and immunoblast

Reaching a diagnosis of KFD promptly is important to limit unnecessary interventions. KFD must be distinguished from other causes of lymphadenopathy and fever such as malignancy, autoimmunity (sarcoidosis, SLE, lupus lymphadenitis), and infection (EBV, HIV, tuberculosis, Bartonella, toxoplasmosis). Histological examination of an excised lymph node is the cornerstone of KFD diagnosis.

Tsang and colleagues concluded that the minimal diagnostic criteria for KFD include the presence of foci, histiocytes, and plasmacytoid monocytes with a paucity of neutrophils, overt infection, or malignancy [6, 8-9] . Lymphoid follicles or follicular hyperplasia can also been seen. Immunohistochemical stains demonstrate CD68+/myeloperoxidase (MPO)+ histiocytes, CD68+/CD123 plasmacytoid dendritic cells, variable CD8+ lymphocytes and immunoblasts [6-10].

Etiology and associations

The etiology of KFD remains controversial. Features of KFD suggest autoimmune or viral etiology. KFD shares many clinical and histopathologic features of a viral infection [6]. Although EBV, human herpesvirus 6, human herpesvirus 8, parvovirus B-19, herpes simplex, cytomegalovirus, and varicella zoster have all been suggested to play a role in KFD pathogenesis, no study has demonstrated clear causality [4, 10, 13-14]. EBV has been examined extensively. Some of the most convincing cases suggesting EBV induced KFD have been in children. Yen et al. describes a 6-year-old boy with diffuse lymphadenopathy, biopsy consistent with KFD, and evidence of active EBV infection based on PCR and immunoperoxidase staining. Others have found no significant difference in the number EBV infected cells in lymphoid tissues of those with KFD compared to controls [14-17]. Some postulate that the absence of EBV infected cells within the lymph nodes does not negate EBV involvement in KFD but rather supports a cytotoxic immune response to EBV infected cells resulting in KFD [15].

Lymph node histology in EBV lymphadenitis differs from that of KFD. Numerous polyclonal plasma cells, absent eosinophils, and CD30+ immunoblasts (seen in our patient) can be seen in EBV lymphadenitis. However, EBV lymphadenitis is associated with significant follicular hyperplasia, Reed Sternberg cells, and an inverted CD4:CD8 ratio [18-20]. In addition, prominent histiocytes and widespread karyorrhexis are not typical of EBV infection.

There are reports of SLE being diagnosed before, simultaneously, or after and KFD diagnosis [3, 5, 8, 10-11]. Interestingly, our patient had a negative ANA that turned positive. It is important to realize that a positive ANA can be seen in the general population and transient elevations are common following viral infections. Lupus lymphadenitis can be difficult to distinguish from KFD. However, the distinctive histiocytes and plasmacytoid monocytes of KFD are lacking in SLE. Plasma cells, hematoxylin bodies, and neutrophils are usually apparent in SLE, but minimal in KFD [7-8]. Our patient was atypical in that she had a significant number of plasma cells on biopsy. The absence of neutrophils, hematoxylin bodies, negative ANA, and self-resolving course make SLE an unlikely diagnosis. However, the association underscores the need for follow up in those diagnosed with KFD.

Given the rarity of KFD, identifying a clear cause is difficult. KFD is likely a transient autoimmune reaction triggered by a nonspecific viral infection in a genetically susceptible host. In our patient, high EBV viral load plus the typical pathological features of KFD suggests widespread necrotizing lymphadenitis triggered by EBV.

Disease course

Kikuchi-Fujimoto disease is generally a benign self-limited disease with a recurrence rate of <5% [3, 6, 8-10, 12]. It is in this authors’ opinion that the benign nature of KFD is essential for diagnosis.

## Conclusions

Kikuchi-Fujimoto disease is a difficult-to-diagnose disease characterized by fever, lymphadenopathy, specific biopsy findings, and benign course. Prompt diagnosis with excisional lymph node biopsy is imperative to diagnose and manage patients appropriately. Therapy is rarely indicated in KFD, and when required is geared towards symptomatic management. Our patient’s biopsy findings and complete resolution of symptoms without intervention supported a diagnosis of KFD in the setting of EBV infection.
